# Interpersonal Psychotherapy for Bereavement-Related Major Depressive Disorder in Japan: A Systematic Case Report

**DOI:** 10.1155/2022/9921103

**Published:** 2022-09-19

**Authors:** Yuko Toshishige, Masaki Kondo, Junya Okazaki, Hiroko Mizushima, Tatsuo Akechi

**Affiliations:** ^1^Department of Psychiatry and Cognitive-Behavioral Medicine, Nagoya City University Graduate School of Medical Sciences, 1 Kawasumi, Mizuho-cho, Mizuho-ku, Nagoya 467-8601, Japan; ^2^Gokiso Mental Health Clinic, 4-5 Ayuchitori, Showa-ku, Nagoya 466-0027, Japan; ^3^MIZUSHIMA HIROKO Mental Health Clinic, 3-12-38 Motoazabu, Minami-ku, Tokyo 106-0046, Japan

## Abstract

Bereavement-related major depressive disorder (MDD) is a common disorder with both mental and physical effects. Specific psychotherapies for bereavement-related MDD remain unavailable in Japan despite its relatively high prevalence. Interpersonal psychotherapy (IPT) is a treatment with established efficacy for MDD, including bereavement-related MDD. There are, however, few studies of IPT for MDD and none at all for bereavement-related MDD in Japan. The efficacy of IPT for bereavement-related MDD needs confirmation in Japanese culture because the expression of emotions during the grieving and mourning process varies across cultures, and the Japanese-specific cultural custom exists of maintaining a relationship with the deceased in the afterlife mainly via a Buddhist memorial tablet, altar, and grave. We present a case study describing the therapist's adaptation of IPT to Japanese culture to treat bereavement-related MDD in a Japanese man with insufficient response to pharmacotherapy who had suddenly lost his mother to heart disease. His mother's death and a dispute with his father both appeared to have contributed to his sustained bereavement-related MDD. The 16-session treatment course for depressive symptoms was monitored using the Beck Depression Inventory-II. Treatment was scheduled weekly, but some sessions unavoidably took place fortnightly because they were conducted in person during the COVID-19 pandemic. The patient's MDD severity continually decreased, functional disability gradually recovered from the beginning until the 3-month follow-up, and the interpersonal relationships with his deceased mother, his wife, colleague, and father changed after IPT. Case studies are inherently limited, but IPT, in consideration of Japanese cultural characteristics for bereavement-related MDD, can be potentially effective in Japan.

## 1. Introduction

Bereavement is one of life's most serious stressors [1]. Grief, the painful response to the death of a loved one, is psychiatrically considered normal, varies in frequency and intensity, and peaks approximately 6 months after the death of a loved one [2]. In some cases, however, bereavement-related depression arises. The boundary between normal and abnormal grief has been a long and continually shifting issue in psychiatry. The Diagnostic and Statistical Manual of Mental Disorders, Fifth Edition (DSM-5) [3] distinguishes the thought content seen in MDD as self-critical ruminations, rather than the preoccupation with thoughts and memories of the deceased seen in normal grief. If guilt is present in normal grief, it typically involves a perceived failing related to the deceased (e.g., not visiting frequently enough). If the bereaved thinks about dying, such thought is generally focused on the deceased and on “joining” the deceased in normal grief, whereas in MDD, such thought is focused on ending one's own life, feeling worthless, and undeserving of life.

In one study, depressive symptoms in bereaved family members were 59% at 1 month, 47% at 3 months, 42% at 6 months, and 39% at 12 months after bereavement [4]. Major depressive disorder (MDD) diagnosed using full diagnostic criteria in bereaved family members was 9% at 4 months and 5.7% at 9 months after bereavement [5]. In Japan, the leading cause of death is cancer, and the prevalences of depressive symptoms and prolonged grief symptoms among the bereaved families of patients with cancer were 22% and 9%, respectively [6]. Furthermore, greater psychological distress, such as bereavement-related depression, increases the risk of physical problems such as cardiovascular and cerebrovascular events [7, 8] and increases mortality risk from many causes, including suicide [7]. Thus, treatment of bereavement-related depression is extremely important because of its high prevalence and its significant negative effects on both the physical and mental health of the bereaved.

Although only a few studies have evaluated psychotherapy efficacy for bereavement-related depression, psychotherapies such as cognitive behavior therapy and interpersonal psychotherapy (IPT) are considered equally effective for MDD in the context of bereavement, as in other contexts [8]. Many therapists around the world have conducted IPT, a psychotherapy with established efficacy for MDD [9], in treating MDD in the context of bereavement. IPT focuses on the relationship between emotions and life events, so IPT manuals have always included complicated grief or bereavement to define treatment of a major depressive episode associated with the death of a loved one. Grief is indeed one of the four interpersonal foci of IPT (the others are interpersonal role disputes, role transitions, and interpersonal deficits/sensitivity).

Grief is the focus of IPT when the depressive episode is linked with the death of an important attachment and the patient is struggling to come to terms with the loss. Both grief and another interpersonal focus may be selected when bereavement-related MDD seems to be sustained not only by a death but also by another interpersonal problem. IPT uses techniques such as therapeutic emotional exploration into current difficult events, validation of those feelings, brief psychoeducation, and communication analysis to help patients solve problems by reconciling gaps of expectations between patients and those around them, improving communication, and building social support [10].

Unfortunately, IPT is rarely offered for bereavement-related MDD in Japan because there are few IPT therapists and the National Health Insurance Plan does not cover IPT. Patients who wish to receive IPT have difficulty finding IPT therapists and must pay the total cost of medical treatment (versus 30% payment if covered by the National Health Insurance Plan). Pharmacotherapy and supportive psychotherapy, which the National Health Insurance Plan does cover, are the mainstays of treatment for bereavement-related MDD. In addition, there are few Japanese IPT studies and no confirmation of the efficacy of IPT for bereavement-related MDD in Japan [11]. The efficacy of IPT for bereavement-related MDD in the context of Japanese culture needs confirmation because views of life and death and the expression of emotions during the grieving and mourning process vary across cultures [12–15], and cultural differences influence the strategies used in IPT.

Japanese culture presents two original strategies that the IPT therapist should consider. First, the therapist can encourage expression of grief and sharing feelings with relatives at Buddhist memorial services, where the patient is more likely to express grief than during typical days, in order to facilitate grieving. In Japan, Shintoism and Buddhism are fused and harmonised, and many Japanese have adopted the Buddhist method when it comes to postbereavement rituals. People mourn, express grief, and gradually accept the death of the deceased while sharing their sorrow with their relatives during postbereavement rituals, such as funerals and memorial services held on the 49th day after death and on the first, second, the sixth, the twelfth, and 32nd death anniversary [16].

Other cultures, too, have formal mourning ceremonies: a funeral, followed by a wake (Irish), shiva (Jewish), or other party or meeting where people can meet, eat, drink, and talk about the deceased. Catholic families can hold special masses (prayers) and light candles for the deceased. In Japan, it is believed that the deceased is reincarnated somewhere in one of six worlds (the world of humans, the world of celestial beings superior to humans, the world of battle-loving demon gods called Ashura, the world of animals and insects, the world of hunger and thirst, or the world of hell) or to emancipation reincarnation to “Gokuraku-Jodo.” Therefore, at regular memorial services, a Buddhist monk recites Buddhist sutras in front of a Buddhist altar and participants join hands in prayer for the deceased to achieve emancipation reincarnation and go to “Gokuraku-Jodo,” a peaceful world without suffering (“heaven”). In Japanese culture, which emphasizes not the individual but group harmony, people read the intentions of others in a group, suppress their own emotions such as anger and grief, act on the same wavelength as the others, and maintain harmony without ever fighting. Therefore, while Japanese people tend to avoid expressing grief, especially in public on ordinary days, they feel that it is psychologically easier to express their grief and share their sorrow with their relatives during cultural postbereavement rituals than in daily life.

Second, the therapist can explore mutual expectations of the patient and the deceased when the patient has a relationship with the deceased in the afterlife and how to adjust expectations when expectations conflict. This approach utilizes the Buddhist memorial tablet, a wooden board placed at the Buddhist altar at home that is considered a temporary home for the soul of the deceased, with the posthumous Buddhist name and the date of death of the deceased, as well as the altar and the grave, for the patient to reestablish a relationship with the deceased. In the Japanese culture of death, people believe that the deceased have not returned to nothingness but exist as a soul in the afterlife and that one can have a relationship with the deceased through the Buddhist memorial tablet, altar, and grave [16]. They are reassured by the thought that the deceased is present in a peaceful state by their sides, watching over them [17].

After “Otsuya,” a ceremony held throughout the night on the eve of the funeral and the day of the funeral, the deceased is cremated, and an urn with the ashes is usually placed in the home until their interment in a grave on the occasion of any of the memorial services. Many Japanese people who have no ancestral grave make not only a Buddhist altar and memorial tablet but also a grave to bury the remains of the loved one, to which they bring offerings and prayers for the well-being of the deceased and their ancestors. Japanese people offer the same food that they eat at the Buddhist altar in the home, where they place a memorial tablet of the deceased and talk to the altar, which is for the deceased and other ancestors, and tablet.

In a survey of Buddhist altar buyers, about 90% of respondents indicated that they had interacted with the deceased in front of the altar, and about 70% interacted with the deceased daily; the average duration of the dialog was 5 minutes. The content of the dialog most often concerned reports of daily life and ranged through thoughts about the deceased, memories of the deceased, consultations, and wishes [16]. Here, we present a case of IPT for bereavement-related MDD (DSM-5), adjusting to Japanese culture.

## 2. Case Presentation

Mr. A, a 42-year-old Japanese male patient, suddenly lost his mother to heart disease. She had lived alone with his father and had suffered from a non-cardiac chronic physical illness for >5 years before her death. The patient had been concerned about his mother's health since she was first diagnosed with the disease and routinely kept in touch with her by phone. Approximately two years before her death, his mother began to lie in bed complaining of poor health, but until the day before she died, she could still go about daily life without major assistance. On her final day, his mother suddenly collapsed on the toilet. Taken to a hospital, she subsequently died.

Mr. A exhibited acute grief. He did mourn privately. He told no one at work about his mother's death, and he mourned so invisibly that they were unaware of his grief. “The hardest thing is that I have no one at all to share my grief with,” “I can't concentrate at work and I'm in a daze at home,” “I have a feeling of emptiness in life,” he said. He felt suicidal and excessively guilty. Specifically, he thought that life was not worth living and actively planned to die; it was not just the thought that death could reunite him with the deceased in the afterlife. Thoughts about the death of the deceased and “joining” the deceased can be part of normal grief, but his thinking involved ending his own life because of feeling worthless, undeserving of life, and too guilty. He had a reduced appetite and insomnia. He kept everything inside, which was a set-up for complicated bereavement. Eight months after the death, he was diagnosed with bereavement-related MDD (DSM-5) at a mental health clinic. On the Beck Depression Inventory-II (BDI-II) [18], his depressive symptom severity score was 43. The patient received antidepressant pharmacotherapy with mirtazapine and escitalopram. Aripiprazole 1.5–3 mg was started as augmentation but immediately discontinued due to akathisia. Two months later, the prescribed medication (mirtazapine 15 mg and escitalopram 10 mg) had only slightly decreased his severe symptoms (BDI‐II = 37).

Mr. A visited our hospital to receive IPT for bereavement-related MDD. He was the only child of a homemaker mother and an employed father. His mother was the primary person with whom he had emotional exchanges and casual conversations. He did not emotionally communicate with his father, who had abused alcohol since the patient's childhood. After graduating from a science graduate school, Mr. A became a company employee. He was married and had two children. His wife provided cooking and cleaning and met his other physical needs but was not emotionally close.

### 2.1. Diagnostic Assessment

Assessed using the Mini International Neuropsychiatric Interview at our hospital, Mr. A was diagnosed with DSM-5 MDD. After careful examination, persistent complex bereavement disorder (PCBD) in DSM-5 Section III (recently superseded by prolonged grief disorder in the Diagnostic and Statistical Manual of Mental Disorder 5^th^ Edition Text Revision (DSM-5-TR) [19]) could not be diagnosed because (1) functional impairment was attributed to MDD and (2) the 12 months required for PCBD diagnosis had not yet passed. His depression, which appeared to have been triggered by his mother's death, worsened whenever he felt alone and sad without his mother, exacerbating his grief.

He stated that his mother was someone he understood so wholeheartedly that no one could ever replace her. He blamed himself intensely for not taking steps to prevent his mother's death, such as suspecting cardiac problems and recommending that she go to a hospital before her death. He thought about her constantly, to the point that it interfered with his work and family life. Meanwhile, he avoided looking at pictures of his mother or visiting places where he had often spent time with her. He constantly avoided expressing and sharing various emotions, including grief. The Japanese cultural tendency to avoid publicly expressing grief in daily life seemed to compound his emotional avoidance, bringing greater levels of grief and depression severity.

Mr. A had to deal with his father's alcoholism and associated problems. His father, left alone after the mother's death, fell after drinking and had to be taken to the emergency room. Mr. A's estranged relationship with his father exacerbated his depression. Thus, his bereavement-related MDD seemed sustained not only by his mother's death but also by a dispute with his father.

### 2.2. Treatment Course

The patient received 16 IPT sessions, each lasting 60 minutes. Sessions were conducted once weekly or once every 2 weeks. The first author (YT) delivered the treatment, having been trained by and in supervision with HM, an International Society of Interpersonal Psychotherapy (ISIPT) certified trainer/supervisor. The patient continued stable doses of mirtazapine 15 mg and escitalopram 10 mg. He completed the 21-item BDI-II self-report measure at baseline, immediately after, and three months after treatment. BDI-II total score ranges from 0 to 63, with higher scores indicating more severe depressive symptoms [18]. A score over 29 is considered severe depression.

The therapist aimed to establish a therapeutic alliance, using verbal and nonverbal communication to establish a rapport with the patient. She conducted IPT for bereavement-related MDD, focusing on grief and the interpersonal role dispute with the father, following a published treatment manual [10].

#### 2.2.1. IPT for Grief

First, to facilitate the grieving process, the therapist encouraged the patient to express how he had felt before and after his mother's death. The therapist did this through encouraging his participation in Japanese postbereavement rituals, such as funerals and memorial services, especially after a mother's death. It was psychologically easier for the patient, who avoided expressing and sharing grief generally, to express grief during Japanese cultural rituals than in daily life. In the rituals, he repeatedly expressed the sadness, pain, and loneliness of not having his deceased mother by his side.

Second, the therapist encouraged the patient to consider a Buddhist memorial tablet, altar, and a grave, through asking him about recent topics relating to his deceased mother. Mr. A was a practicing Buddhist and the therapist integrated Buddhist spiritual beliefs that the deceased exist as a soul in the afterlife and that one can have a relationship with the deceased through the Buddhist memorial tablet, altar, and grave into the sessions in order to help the patient deal with grief. He decided to make the mourning implements. The therapist explored with the patient his and his mother's mutual expectations regarding the design of the tablet and altar and the location of the grave. Mr. A reported that his deceased mother never replied to him directly anymore and said he would like to reestablish a relationship with her.

Although there were no communication problems with the mother, his strong emotional avoidance made it appear as if there was a gap in expectations in that he had not prepared a tablet, altar, and grave. Mourners generally prepare a tablet and altar by the 49th Buddhist memorial service, and a grave is generally prepared by the 49th Buddhist memorial service or the first or second death anniversary. Therefore, in sessions, in addition to eliciting his expectations of his mother, the therapist used emotional encouragement to elicit her mother's expectations of him in his mind. Mr. A and his mother had not discussed mutual expectations they had about the tablet, altar, and site of her grave before her death. However, Mr. A and his mother had talked about their feelings and mutual expectations about a variety of matters, important and trivial, since childhood, so he felt that over time, they gradually came to know each other's feelings and mutual expectations without exchanging words. Therefore, he could imagine in his mind his mother's expectations of him about the tablet, altar, and gravesite through this treatment. Mr. A thought that his deceased mother expected him to have gentle designed tablets made and to have her grave in a place where she could see her favorite view. Mr. A wanted to live up to his beloved mother's expectations, and the therapist and the patient confirmed that doing these things would resolve any gap in expectations.

He then chose a pleasant, caring design for the Buddhist memorial tablet that evoked a gentle feeling his deceased mother would have liked. He decided to place her grave near his house, which had a view of the landscape she loved, where he could frequently visit with her and her soul could see her favorite view. In Session 10, he said, “Her bones are alone in the grave, but her soul is not lonely and is spending time with those close to her [her deceased father and deceased dog]” when describing having placed his deceased mother's ashes in her grave. He then said that he felt at peace. In Session 11, he felt his deceased mother was close to him and felt calmer when he placed his hands on the home altar with the Buddhist memorial tablet in the morning and evening. He seemed to find a deeper level of comfort in his mother's death and to have reestablished a warm relationship with her.

Third, to enhance social support and counter his depressed social withdrawal, we explored the possibility of having emotional exchanges and casual conversations with his wife or others in order to reestablish interests and relationships. He mentioned having previously felt fulfilled by casual conversations with a close older colleague who had taught him interesting things, and he had felt relieved when talking with that colleague after his mother's death. However, the COVID-19 pandemic precluded frequent in-person meetings with his colleagues. We explored the possibility of talking with his colleague via email and Zoom. In Session 9, Mr. A said he wanted to have a chat with his colleague that was not really work-related (about his colleague's hobbies, his colleague's interesting acquaintances) and could text his colleague to see if they were both in the office on the same day, although office work days were quite low because of COVID-19. Mr. A reported feeling a positive change in his mood after briefly chatting with his colleague during long company Zoom meetings.

Further, the therapist helped Mr. A to explore developing an emotional communication with his wife. It became clear that his wife was a logical thinker and he primarily expected physical support from her. His wife took the initiative to look after the children herself when he was very tired and encouraged him to take morning walks to get fit. He did not fully share his grief over his mother's death with his wife, but he appreciated her concern for his well-being and care for their children when he was not feeling well and wanted time alone. This reduced his anxiety in his relationship with his wife.

As already described, the therapist encouraged Mr. A to facilitate the grieving process while reestablishing a relationship with the deceased mother in the afterlife. When talking about his mother, he did not cry in sessions, but he looked teary-eyed, his speech paused, his facial expression stiffened, and he always made a strong gesture with fists and rubbing his hands. His hard facial expressions and hand gestures, which appeared to be signs of anxiety, gradually improved towards the end of sessions. The therapist did not assign homework: these changes in expression appeared a loosening up of affect with growing trust in the therapeutic relationship.

#### 2.2.2. IPT for the Interpersonal Role Dispute

The patient had been coping with his father's problematic alcoholic behavior alone after his mother's death. He had previously managed this with his mother until he graduated from high school but had not had to do so since entering university in the city where he could pursue his dreams living far from his parents. The patient was angry when his father suddenly again become an unwanted burden for him after his mother's death. There is an expectation that Japanese children care for their widowed parent when the parent is ill, whatever the illness. His father had been admitted to a facility for alcohol treatment one month before the IPT sessions began.

Concerned that his father's drinking might bother others, Mr. A maintained frequent contact with his father and tried to persuade him not to drink. Unfortunately, each contact with his father worsened his depression, who ignored Mr. A's concerns and kept drinking. Mr. A wanted to separate from his father, but the cultural expectation to care for the widowed parent made him guilty. He felt both guilt and strong anger towards his father and recognised that it was natural to have these feelings. However, because he rationally suppressed some of his feelings toward his father, he did not yell at him. Nor did he share his feelings with those around him, including his wife. The dispute was in the stage of dissolution.

The therapist explored with the patient how his father was coping with the loss of his wife, considering the possibility that his father's alcohol use might have increased to drown his sorrow. In fact, his father had rarely spoken to his mother since long before her death, and his attachment to her appeared to be so poor that he did not consider conducting the mother's Buddhist rituals even after her death. These require quite burdensome tasks for the bereaved family: to plan for holding a funeral or memorial services for the deceased and meet with funeral directors and Buddhist monks, but his father did not try to plan for it; he only participated because Mr. A told him to. Thus, the father's grief appeared not as profound as Mr. A had imagined, and his drinking did not obviously increase following the death.

The therapist explored the mutual expectations of the patient and his father and used communication analysis regarding his father's alcohol consumption. Worried that his father's drinking would bother others, Mr. A candidly asked for sobriety. His father rarely expressed his feelings in words, but he seemed to want his son to leave his drinking alone and secretly bought alcohol. The father never met the patient's expectations.

In order to end the relationship between him and his father, the therapist explored with the patient whether he could expect his father to recover from alcoholism and become sober in future. The person's motivation for treatment is crucial for alcoholism prognosis, yet unfortunately, his father had lacked that motivation for years and seemed unlikely to improve. The therapist helped Mr. A talk to his wife about his concern over his father's alcoholism using role play. She advised him to leave his father in the care of the treatment facility so as not to burden the patient. The therapist encouraged Mr. A to ask someone at the facility whether his father was bothering others with his drinking. He was relieved to learn that despite his father's problematic drinking, people at the facility did not consider it a nuisance and were doing their job to cope with it.

Hence, Mr. A was able to adjust his expectations of his father's sobriety—he no longer expected sobriety—and felt he could leave his father under the care of the institution. It helped that his wife respected his emotions. He no longer felt overly responsible for his father's alcoholism, as his father never wanted to stop drinking. His depressive symptom severity no longer progressed because of his relationship with his father.

### 2.3. Treatment Termination and Outcome

During treatment termination, Mr. A said: “I have learned to accept the fact that everyone will die one day… I am getting used to life without my mother,” “I feel a little calmer now that I have a Buddhist altar,” and “I have started to think about watching a variety TV show.” The BDI-II, decreasing from 37 (pretreatment) to 23 (posttreatment), supported the patient's perspective of improvement. Although Mr. A still scored in the moderately depressed range on the BDI-II, his appetite and insomnia were improved and he was no longer suicidal or overly guilty. That is, he no longer seemed to be depressed, but rather very sad.

In this treatment, the therapist continued to explore mutual expectations between himself and his deceased mother regarding the relationship with his father. He expressed anger towards his mother at the 2-month follow-up, saying that he had long encouraged his mother to divorce his father, but that she had tolerated him despite his alcoholism, had been unable to divorce him, and had gotten physically ill as a result. At 3-month follow-up, he had started studying English to assist him in his future work. His father had had to be rehospitalized, but he felt he could trust the facility with the hospitalization procedures and felt less psychological burden than before. His BDI-II decreased from 23 to 18 from posttreatment to the 3-month follow-up.

## 3. Discussion

This case study implemented IPT for a Japanese patient who exhibited bereavement-related MDD and had only a limited pharmacotherapy response, focusing IPT on both grief and a paternal interpersonal role dispute [10], involving the therapist's consideration of Japanese culture. Depressive severity continuously decreased until three months after IPT completion, with BDI score dropping from the very severe (37) to the mild (18) range. His functional disability recovered, and his motivation to work increased three months post-IPT. Our results suggest that IPT may be adaptable to and effective for bereavement-related MDD in Japan.

Why did depressive severity decrease so substantially? First, his bereavement-related MDD seemed sustained not only by his mother's death but also by the resurgent dispute with his father that her death entailed. The therapist therefore conducted IPT for bereavement-related MDD focusing on both grief and on his paternal interpersonal role dispute, following the IPT treatment manual [10]. Life is much the same around the world in that death is both a bereavement and often a trigger for family conflicts. In Japanese culture, where family harmony is paramount, families often maintain apparent harmony even when they do not get along, as other family members manage to reconcile antagonists. As in this case, the death of a family member who had managed to reconcile family members often brings the dispute to the surface as contact between no longer buffered surviving family members increases. Focusing on the two interpersonal problem areas, grief and interpersonal role dispute, facilitated the patient's grieving process and rebuilt his interpersonal relationships with his deceased mother, his wife, and his colleague, while resolving the patient's dispute with his father.

In focusing on grief, the therapist adapted two original strategies in observance of Japanese culture with respect to bereavement. These may well have facilitated the grieving process by reestablishing a relationship with the deceased, reducing depression severity. In the process of facilitating the grieving process, therapeutic emotional exploration based on the therapist's consideration of Japanese-specific culture enabled the patient to feel emotions such as sadness, pain, loneliness, anger, and guilt associated with bereavement.

In the process of reestablishing a relationship with the deceased mother in the afterlife, exploring mutual expectations in the patient and the deceased mother enabled him to adapt to the change from a relationship between the living to a relationship between the living and the dead (a presence by his side to watch over him) and to reestablish a relationship with the deceased mother. Indeed, in Session 11, he felt his deceased mother was close to him and felt calmer when he touched the altar. During treatment termination, he noted having “learned to accept… that everyone will die one day.” We think integrating Buddhist spiritual beliefs into IPT is a useful modification for some Japanese patients. These original strategies incorporating Japanese culture into IPT treatment of bereavement may well have contributed to reducing depressive severity. IPT always seeks to adjust to cultural context [20].

In typical IPT for grief, the therapist encourages the patient to talk about not only positive but conflictual, negative feelings the patient may have towards the deceased in order to help the patient view the deceased and the relationship with greater balance. In Asian cultures, criticism of the dead is not well regarded. Therefore, the therapist did not encourage the patient to discuss his negative/ambivalent feelings towards his deceased mother but explored with the patient mutual expectations between them and adjusted expectations where there were discrepancies, which enabled him to see his deceased mother in a balanced way. In fact, the patient was able to express anger towards his mother, albeit two months after the end of treatment.

Mr. A had wanted his deceased mother to divorce his father, but she wanted him to allow her to stay somehow without divorcing him. She had been a housemaker for a long time and, like Mr. A, might have wanted a divorce but have feared that financial problems made divorce difficult. Reflecting on this, Mr. A seemed able to understand her not divorcing his father, which resolved discrepancies between him and her. This was also thought to contribute to reduction in depressive severity. In addition, he also was able to express anger about his father, which may have helped get some of the anger toward his mother off his chest.

When focusing on grief and the interpersonal role dispute, the therapist supported Mr. A's relationships with his wife and colleague to rebuild, increase security, and reduce his loneliness. She encouraged him to share with his wife the problems associated with his father's alcoholism and to have the kind of casual conversations with his colleague that he had previously had with his mother. Numerous studies have shown an association between low social support and depression symptoms and diagnosis [21, 22]. A previous study revealed that loneliness is a risk factor for depression [23]. Among social supports, intimate relationships (e.g., marriage) are particularly important [24]. In this IPT treatment, the patient gained a more secure relationship with his wife and close colleague, increasing social support and decreasing the loneliness, which seemed to contribute to depressive symptom improvement.

This is a case report. Limitations of generalization, methodological rigor, and objectivity may be present. We look forward to further accumulation of evidence relating to therapies for bereavement-related MDD in Japan. The case report suggests that IPT for bereavement-related MDD with the therapist's consideration of Japanese culture, which can focus on two interpersonal problem areas, such as grief and the interpersonal dispute, can be a fruitful treatment in Japan.

## Data Availability

Data sharing is not applicable to this article as no datasets were generated or analyzed during this study.
